# Artificial intelligence for precision oncology: beyond patient stratification

**DOI:** 10.1038/s41698-019-0078-1

**Published:** 2019-02-25

**Authors:** Francisco Azuaje

**Affiliations:** 1 Bioinformatics and Modelling Research Group, Department of Oncology, Luxembourg Institute of Health (LIH), L-1445 Strassen, Luxembourg; 2 Present Address: Computational Biomedicine Research Group, Center for Quantitative Biology, Luxembourg Institute of Health (LIH), L-1445 Strassen, Luxembourg

## Abstract

The data-driven identification of disease states and treatment options is a crucial challenge for precision oncology. Artificial intelligence (AI) offers unique opportunities for enhancing such predictive capabilities in the lab and the clinic. AI, including its best-known branch of research, machine learning, has significant potential to enable precision oncology well beyond relatively well-known pattern recognition applications, such as the supervised classification of single-source omics or imaging datasets. This perspective highlights key advances and challenges in that direction. Furthermore, it argues that AI’s scope and depth of research need to be expanded to achieve ground-breaking progress in precision oncology.

## Introduction

Artificial intelligence (AI), a field of computer science whose origins can be dated back to the 1940s,^1^ aims to develop computational systems with advanced analytical or predictive capabilities. Such systems are typically designed to solve complex data-intensive problems that require the prediction of, or reasoning about, their underlying phenomena. Machine learning (ML) represents the most successful branch of AI. ML is concerned with the development of programs with the capacity to learn from data. Such a learning capacity is achieved by incrementally improving on a prediction task based on problem-specific measurements of performance.^2^ As defined by Tom Mitchell more formally: “A computer program is said to learn from experience *E* with respect to some class of tasks *T* and performance measure *P*, if its performance at tasks in *T*, as measured by *P*, improves with experience *E*”.^3^

ML has a solid history of applications in biomedical research,^4–6^ and is becoming a driver of pre-clinical and clinical oncology research.^7,8^ Over the past few years, ML’s potential in precision oncology has become more apparent by the reporting of major advances in deep learning (DL), and its application to a variety of diagnostic, prognostic, and other predictive tasks.^5,9–11^

DL is a sub-branch of ML that comprises a diverse family of computational models consisting of many (deep) data processing layers for automated feature extraction and pattern recognition in large datasets, e.g., multilayer feed-forward neural networks.^12,13^ DL’s advances in cancer research have been made possible not only by the availability of larger datasets and accelerated computing capabilities, but also by developments in statistical learning theory, algorithms, and open-source software accumulated over the past 4 decades.^13^

The current visibility of DL in precision oncology has been in large part due to its impressive performance in classifying imaging data in different clinical domains. Examples include: the detection and classification of skin lesions,^11^ the identification and categorization of lung cancers,^14^ and the detection of metastases in women with breast cancer,^15^ all of which apply different versions of a DL technique known as convolutional neural networks (CNN).^12,16^

Precision oncology research also benefits from a variety of alternative ML approaches for supervised and unsupervised pattern analysis in datasets originating from multiple sources, including tumor-derived omic profiles. This includes, for example, the prediction of oncogenes and tumor suppressors with random forests.^17^ Key examples of (non-DL) ML techniques include: probabilistic models, kernel-based models (e.g., support vector machines), and decision tree-based models (e.g., random forests and gradient boosting machines; GBM).^3,18,19^ These and other approaches have provided the basis for promising predictive modelling applications in oncology research.^20,21^ More detailed examples are provided in the following sections.

**Fig. 1:**
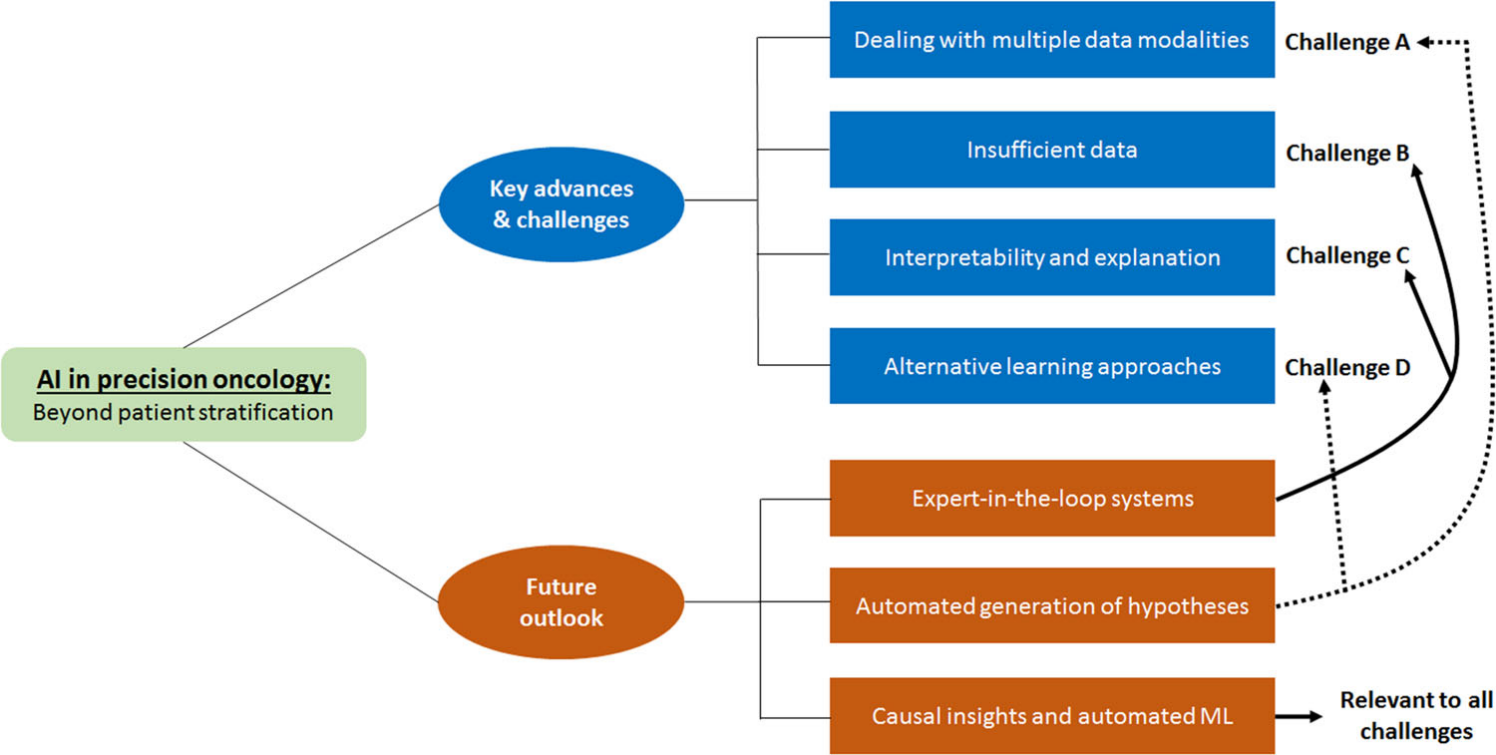
AI in precision oncology: beyond patient stratification. Selection of key advances and challenges, as well as long-term outlook, discussed in this perspective. Associations between future outlook and challenges are indicated with arrows connecting the former to the latter

To date, ML has played a prominent role in facilitating novel applications that mainly rely on the supervised identification, correlation, and classification of complex data patterns for patient stratification. However, to deliver on the promise of a more precise prevention, detection, and treatment of cancers, other clinically-oriented computational modelling challenges must be tackled. This perspective underlines a selection of such research challenges or requirements for moving the field forward. It argues that ML offers, yet to be fully tapped, opportunities for enabling precision oncology far beyond relatively well-known applications, such as the supervised classification of single-source omics or imaging datasets. Moreover, there is a need for modelling approaches that can assist researchers and clinicians in better understanding biological causality. To advance and accelerate precision oncology research, the scope of questions and applications that AI can address ought to be considerably expanded (Fig. 1).

This article discusses four key challenges in ML for precision oncology: dealing with multiple data modalities, insufficient data, interpretability and explanation, and alternative learning approaches. Each section begins with overviews of significant progress achieved to date. The article concludes with an outlook, outstanding questions, and final remarks.

## Challenge A—dealing with multiple data modalities

Precision oncology research increasingly relies on the integrated analysis of multiple types of omics data, e.g., transcriptomics and proteomics. ML algorithms and applications have been developed to automate different aspects of such a process at different levels of integration.^22^ This includes the combination of data at the model’s input level, the combination of features extracted from multiple data sources independently and of predictions made by different ML algorithms.^23^

Recent examples relevant to precision oncology are cancer sub-typing based on the unsupervised learning of patterns observed across point mutation, copy number, methylation, and RNA expression datasets;^24^ the prediction of several clinical outcomes (e.g., survival outcomes) of ovarian cancer patients through the combination of copy number aberration, epigenome and transcriptome datasets using a model that extracts predictive patterns from both labeled and unlabeled samples;^25^ and the prediction of glioblastoma progression based on the integrated analysis of DNA methylation and matched imaging data.^26^

Although these are important advances, there is still a need for expanding the range of predictive integration, in terms of data types and ML algorithms. An important challenge is the prediction of patient outcomes or phenotypes based on the integrative analysis of multi-omics, imaging, and other types of clinical data. Examples of progress in this area are the prediction of survival of lung cancer patients using transcriptomics and histopathology data;^27^ and the analysis of correlations between magnetic resonance-derived features, gene expression and patient survival data.^28^ Despite such notable progress, additional data types and ML algorithms remain to be deeply investigated. One way to improve such approaches is to enhance the integration of image-derived information with additional layers of omics data and expert-defined annotations. Moreover, future model assessments should include additional independent databases with images representing richer and more complex patterns beyond those provided by well-established datasets, e.g., the TCGA image collection.^27^

The application of ML to multiple data modalities will depend on the availability of sufficiently large, matched, and carefully annotated datasets. Future applications will benefit from ML strategies that are suitable for dealing with relatively small datasets (next section). Moreover, meaningfully linking multi-omics to imaging data for predictive modelling will also depend on advances in sample extraction and processing technologies. A crucial challenge is the spatially-accurate matching of in vivo imaging and ex vivo data.^29^

## Challenge B—insufficient data

ML in general, and DL in particular, rely on large collections of data. For example, outside cancer research, typical DL systems are trained with several thousand or even millions of samples previously labeled by humans.^2^ Despite the continuing reduction of the costs of data generation, including molecular readouts using different technologies, the access to massive high-quality datasets in precision oncology will be constrained. This challenge involves a relative lack of large truth or reference datasets containing, for example, carefully molecularly characterized tumors and their corresponding detailed clinical annotations.

To overcome this obstacle, researchers can apply *transfer learning* (TrLe) methods, which have been developed for different ML algorithms and applications since the 1990s.^30^ TrLe’s main assumption is that predictive features learned in an application *domain X* can in principle be applied to a different, but related, application *domain Y*.

A prominent example of TrLe in precision oncology is the automated classification of skin cancers with DL, as reported by Esteva et al.^11^ Their classification system consisted of a deep CNN, which was pre-trained on millions of images representing more than 1000 generic image classes (the GoogleNet Inception CNN). CNNs represent a diverse family of multi-layer artificial neural networks, which are typically applied to the classification of images. A CNN usually consists of multiple layers of data filters applied to different regions of an image. Such filters are useful for automatically extracting features correlated with the content of the image, e.g., filters specialized in detecting edges and filters specialized in extracting higher-level abstract attributes. The GoogleNet Inception model is a widely-applied CNN model, which was originally trained for accurately classifying millions of generic images stored in the ImageNet database.^31^ Such a generic DL system provides the architecture and learning parameters needed to recognize features that are commonly relevant across image processing problems, e.g., segmentation and texture features. Next, Esteva et al. fine-tuned the system using more than 100K skin lesion images associated with more than 700 skin diseases. On hundreds of skin cancer images, they showed prediction performances comparable to those achieved by expert dermatologists.

More recently, Coudray et al.^14^ applied a similar TrLe strategy, also based on the GoogleNet Inception CNN, to predict lung cancer subtypes using hundreds of whole-slide images. They reported a prediction performance comparable to that obtained by pathologists. The potential of TrLe beyond cancer imaging has also been demonstrated. For instance, Sevakula et al.^32^ improved cancer classification using gene expression data and a TrLe strategy based on a different type of DL technique, called stacked autoencoders. In their study, autoencoders were used for: (1) generating compressed data representations that are found in common across different cancer datasets; and (2) using such extracted features to initialize the parameters (network weights) of a DL model.

Apart from DL, TrLe can also be implemented with different ML models and smaller datasets. Turki et al.^33^ presented an alternative TrLe-based approach that offered improved prediction of drug sensitivity of multiple myeloma patients by transferring patterns learned in datasets from patients with breast and lung cancers. A method related to TrLe, multi-task learning, was also shown to make accurate predictions of drug sensitivity of cancer cell lines.^34^ This strategy allowed a knowledge exchange between two different, but complementary, tasks: dimensionality reduction and classification of gene expression datasets.

To address the challenge of dealing with small datasets, there are also opportunities beyond TrLe approaches. For instance, outside precision oncology, the implementation of model building strategies based on multiple training runs and surrogate datasets has led to reproducible and clinically-relevant predictions, which can be as accurate as those obtained from models trained on much larger datasets.^35^ Shaikhina and Khovanova,^35^ for example, reported a neural network model that accurately and robustly predicts the strength of bones in osteoarthritis patients using training datasets as small as 22 samples. They overcame such small sample limitations by implementing multiple runs of thousands of models trained simultaneously with different learning parameters. The resulting models were collectively evaluated, and the highest performing model was selected for further testing. To complement this training strategy, they also generated surrogate datasets to expand the amount of data available for testing the models. Such surrogate datasets represent noisy versions of the original data, and are useful for supporting the evaluation of the trained models in situations when additional data are not available for model testing.

## Challenge C—interpretability and explanation

The predictive performance, e.g., the accuracy or precision, of a ML system is a critical, but by no means the most important prerequisite for its deployment in the clinic. Another key requirement is a ML system’s capacity to make predictions that are sufficiently understandable or interpretable to humans. Typically, the higher the complexity of a model the harder the interpretability challenges posed by such models. The problem of *black box* models is not limited to DL, but also concerns relatively less complex models with more user-friendly model representations, such as random forests, which often comprise hundreds of decision trees.

Despite ongoing efforts in biomedical and healthcare research, such as those reported in refs. ^36–38^, the development of methods for interpreting ML models is at a relatively early stage, in particular in the field of precision oncology.^39^ Interpretability can be achieved at different levels of data processing and abstraction, and may not be necessarily constrained to specific models. Augmenting the interpretability of ML approaches will allow users not only to peer into a model and understand how predictions are made, but also, perhaps more importantly, to obtain explanations for patient-specific predictions. This may necessitate, in some cases, a general view of which prediction features (e.g., molecular biomarkers or image features) are important to assign a patient to a particular clinical outcome. Other problems may need more detailed and graphical depictions of evidence relationships underpinning individual predictions.

Outside oncology research, there are several examples of the potential of interpretability embedded into ML systems. Among them, a system that predicts the risk of hypoxemia and offers explanations of the underlying most relevant risk factors in real time;^40^ the explanation of object identifications made by a DL system based on the similarities between the image being processed and previously analyzed prototypical images;^41^ and the automated identification of image regions that match with parts found in reference images used for training the model.^42^

Although domain-specific advances are still needed, DL models are becoming more interpretable through the use of several attribution methods. Such methods estimate the relevance, or contribution, of each input feature in a DL network (e.g., pixel regions) for making a specific prediction (e.g., image classification).^43^ Encouraging progress is also being achieved beyond DL, such as in the case of random forest models. Relevant examples include: the estimation of the importance of input features (Gene Ontology terms) for the prediction of gene expression states in the aging brain,^44^ and the extraction of genomic interactions for explaining gene expression patterns observed in different model organisms.^45^

A key advantage of the examples outlined above is their relative flexibility and ease of adaptation in different modelling tasks relevant to precision oncology. However, precision oncology lacks sufficient tools with demonstrated capacity for offering a deeper or novel understanding of the biological mechanisms behind patient-specific predictions. This is particularly challenging when considering the other problems highlighted in this perspective. Despite the usefulness of “agnostic” or global approaches to interpretability, a new generation of methods adapted to the particular needs and questions of precision oncology is warranted. Yu et al.^46^ argue that the interpretability of models in biomedical research will be improved with “visible approaches”. Such approaches are based on the use of prior biological knowledge of the biological structure of cells and tissues for guiding the design of ML systems. Advances in this area will not only increase the acceptability of ML in the lab and clinic, but also it will aid in the generation of new hypotheses (e.g., biomarkers and therapeutic targets) and in understanding the mechanisms underlying particular pathological states. A potential limitation of this approach is that in different domain-specific applications such prior knowledge may not be readily available, and additional efforts may be required for its acquisition and computer-readable representation.

## Challenge D—alternative learning approaches: hybrid models and reinforcement learning

Other ML approaches, based on adaptations of those highlighted above, are also applicable and useful for precision oncology research. Among them, there is a diversity of strategies that either bag or boost groups of relatively weak models for jointly achieving better prediction performance. Boosting and bagging can be implemented by combining, for example, different sets of decision trees (as in the case of GBM) or neural networks (as in the case of network ensembles), respectively.

Reinforcement Learning (ReLe) is a ML branch that has not been yet widely investigated in oncology research. ReLe-based models learn to reach better predictive performance by a series of trial and error steps. Essentially, the model continually senses its environment and processes the resulting feedback, which includes rewards assigned to each learning trial, until an optimal performance is obtained. Yauney and Shah^47^ demonstrated the potential of ReLe for predicting optimal dosing regimens to reduce brain tumor size in clinical trials. Their model learned temozolomide (TMZ) and procarbazine, 1-(2-chloroethyl)-3-cyclohexyl-l-nitrosourea and vincristine (PCV) dosing regimens. The latter was estimated based on the reduction of mean tumor diameters. Moreover, using simulated clinical trials, their model reduced dosage concentrations and the number of treatment cycles in comparison with values produced by clinical experts. Despite such encouraging progress, an important challenge is to evaluate these systems in larger independent patient cohorts, as well as deeper investigations involving different cancers and treatments.

ReLe-based approaches and their combination with DL models will be increasingly applied to precision oncology. Recent examples include: a deep reinforcement learning algorithm for the early detection of pulmonary nodes in computed tomography images;^48^ a ReLe model for controlling chemotherapy dosing in cancer patients;^49^ and a deep reinforcement learning for the estimation and adaptation of radiation protocols for lung cancer patients.^50^ Although these and other examples reviewed in detail by Mahmud et al.^51^ provide evidence of the potential of ReLe in precision oncology, deeper investigations involving different and larger patient cohorts will be needed. Another key requirement is the incorporation of multiple data modalities associated with different cancer types and therapeutic strategies.

## Future outlook and concluding remarks

Based on the progress and challenges outlined above, this section offers a brief selection of ideas for further advancing and accelerating the application of ML in precision oncology.

ML will continue moving precision oncology research forward and closer to the patient. Nonetheless, such efforts will require oncology and AI researchers to address the gaps and challenges outlined here and elsewhere.^2,5^ New ways of thinking about AI will also be necessary to go well beyond incremental advances in pattern recognition capacity. Such steps will be decisive to substantially demonstrate the value of AI for bringing better preventive, diagnostic, and treatment options to patients.

A key question is how to get oncology and AI experts efficiently involved in the different stages of the ML development cycle. This issue is particularly relevant to Challenges B and C (Fig. 1). Such expert-in-the-loop systems may improve not only the way input datasets are selected and predictive performance is evaluated, but also they could guide the learning process.^52^ The latter may comprise techniques for dynamically capturing users’ feedback and its incorporation into the adaptation of the model, e.g., its architecture and learning parameters. Depending on the application domain, users may consist of a combination of clinical- and computationally-minded experts, e.g., pathologists and ML engineers working together to fine-tune models. Apart from developing approaches for giving clinicians feedback on model predictions, there are opportunities for incorporating expert knowledge during model design or prior to prediction interpretation. Outside precision oncology research, examples of strategies in this direction have been reported by Yu et al.^46^ and Girardi et al.^52^ The latter demonstrated the active role of medical experts at different stages of a data analysis workflow in the setting of cerebral aneurysms research. Such a modelling strategy allowed experts to select the data and metadata necessary for addressing specific research questions. Moreover, based on interactive visualizations, the expert could choose multiple combinations of parameters for building a classification model.

Another area that merits further investigation is the development of ML systems that can extend their main, originally-intended application purposes. This issue is of particular relevance to Challenges A and D. This could include, for example, using a cancer diagnostic system for suggesting hypotheses about the interplay between the underlying diagnostic biomarkers and candidate therapeutic targets associated with a specific patient group. This may require computational agents working in the background to discover novel clinically-meaningful associations between molecular profiles and other clinical variables stored in medical records. This can be useful to boost and accelerate: (a) the identification of clinically-relevant subtypes in clinical trials; (b) the discovery of novel biomarkers or connections between them and clinical outcomes; and (c) the identification of mechanistic insights for guiding therapeutic strategies, which can then be fed back to researchers.

Can we give computational systems the ability to infer testable causality? This will continue representing one of the central questions for AI research and its application in and beyond biomedical research. Addressing this question will impact different aspects of ML research for precision oncology, such as those concerning Challenges A and C. Satisfactory answers to that question, even in very specific and controlled environments, will not be feasible through the straightforward application of existing ML algorithms or their incremental extensions for simply achieving greater accuracy or computing efficiency. Advances in this field will also continue benefitting from ground-breaking progress previously achieved in the field of probabilistic reasoning.^53^ An example of progress related to this challenge was recently provided by Yoon et al.,^54^ who proposed a model that can estimate individualized treatment effects based on the analysis of counterfactual clinical outcomes.

It will be indispensable to endow AI with novel capabilities to achieve ground-breaking progress in predictive generalization. Thus, advances in this direction should go far beyond an improvement in prediction accuracy or reproducibility in independent datasets from similar patient populations. It will require AI systems that can generalize across settings in which data distributions, potential sources of bias, and predictive performance objectives vary. Another step forward for aiding researchers in addressing this challenge is the development of ML pipelines that automate the design and evaluation of algorithms (relevant to Challenges B and D). This is a crucial step not only for facilitating and accelerating the implementation of models, but also for delineating to the clinician the reasoning underlying the model predictions, as exemplified by the AutoPrognosis system by Alaa and van der Schaar.^55^

This article provided a perspective on key progress and challenges in ML for precision oncology research. In particular, it focused on four such areas concerning: dealing with multiple data modalities, insufficient data, interpretability and explanation, and alternative learning approaches. Based on such challenges and recent progress, it briefly presented recommendations for moving the field forward.

To improve and democratize precision oncology, the scope of questions and applications that AI can address needs to be expanded. This will further augment, without replacing, the analytical and decision-making capacity of human experts. Regardless of whether most of these and emerging challenges can be overcome, one thing is certain: ML will transcend its main role as a toolbox for accelerating pattern classification in pre-clinical and clinical research.
